# Supplementary material for: Cost effectiveness of temporary isolation rooms in acute care settings in Singapore
Source: PLoS One. 2022 Jul 22;17(7):e0271739. doi: 10.1371/journal.pone.0271739 (PMC9307192; doi:10.1371/journal.pone.0271739)
Supplement: S1 Appendix — (DOCX) [file pone.0271739.s002.docx]

Appendix

Example of the R script and output used to estimate attributable mortality

> sepsishai <-subset(r_hai_clean_final,haisepsis==1) # subsetting patients with sepsis HAI only (n = 142)

> table(sepsishai$state.allcausedeath) # presenting 2 by 1 table

0 1

105 37 # data generated for table 1

> prop.test(x=sum(sepsishai$state.allcausedeath), n= length(sepsishai$state.allcausedeath),conf.level = 0.95) # proportion of deaths in patients with sepsis HAI # one proportion z test with 95% CI

1-sample proportions test with continuity correction

 data: sum(sepsishai$state.allcausedeath) out of length(sepsishai$state.allcausedeath), null probability 0.5

X-squared = 31.613, df = 1, p-value = 1.882e-08

alternative hypothesis: true p is not equal to 0.5

95 percent confidence interval: # data generated for table 2

0.1922298 0.3421151

sample estimates: p = 0.2605634
